# Tinea labialis in a patient with Schnitzler syndrome on interleukin-1 receptor antagonist

**DOI:** 10.1016/j.jdcr.2025.08.004

**Published:** 2025-08-22

**Authors:** Nathan Mooney, Arshpreet Grewal, Susan Pei, Paul Bogner, Drew Kuraitis

**Affiliations:** a Jacobs School of Medicine, Buffalo, New York; b Department of Dermatology, Roswell Park Comprehensive Cancer Center, Buffalo, New York; c Department of Pathology, Roswell Park Comprehensive Cancer Center, Buffalo, New York; d Department of Dermatology, Tulane University School of Medicine, New Orleans, Louisiana

**Keywords:** anakinra, dermatophyte, fungal infection, interleukin-1 receptor, Schnitzler’s syndrome, tinea labialis

## Introduction

Schnitzler syndrome (SS) is a rare autoinflammatory disease characterized by a chronic, nonpruritic urticarial rash and monoclonal gammopathy, driven by excessive IL-1 signaling.^1^ In addition to skin involvement, patients frequently experience recurrent fevers, arthralgia, arthritis, and bone pain. If left untreated, SS can lead to severe complications, including AA amyloidosis and an increased risk of hematologic malignancies. One of the first-line treatments for SS is anakinra, an interleukin (IL)-1 receptor antagonist that effectively controls symptoms but does not cure the disease.^2^ Common side effects of anakinra include pruritus, headaches, and an increased risk of bacterial infections.^3^ Superficial fungal infections, such as tinea, occur when dermatophytes are able to attach to and grow within keratinized tissue. Tinea rarely presents on mucosal surfaces of immunocompetent individuals, presumably due to lack of substrate to host fungal organisms. Herein, we present a patient with SS treated with anakinra who developed a case of tinea labialis, suspected to be due to IL-1 blockade.

## Case report

**Fig 1:**
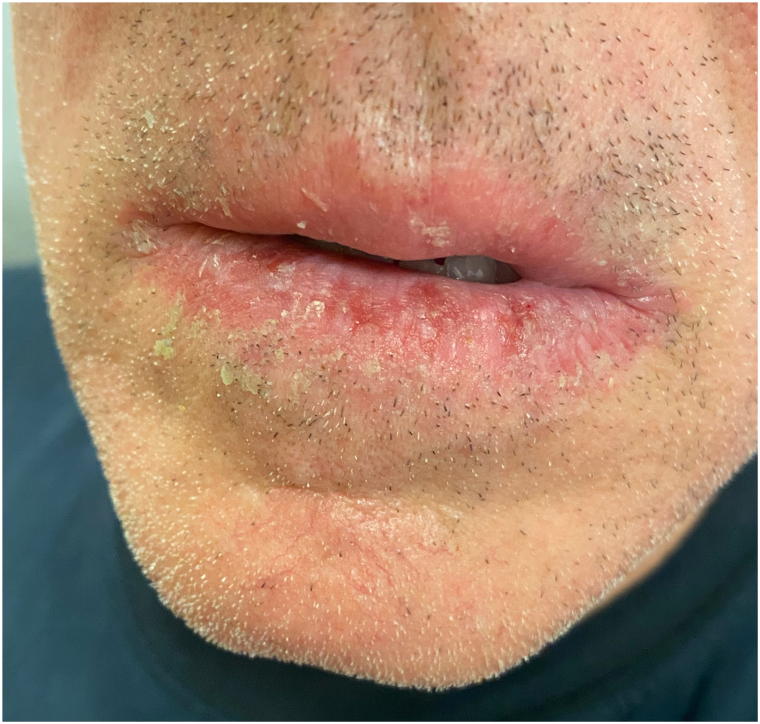
Clinical presentation of tinea labialis. Erythema, scaling, and shallow erosions of the vermillion lip, with crusting to the vermillion border.

**Fig 2:**
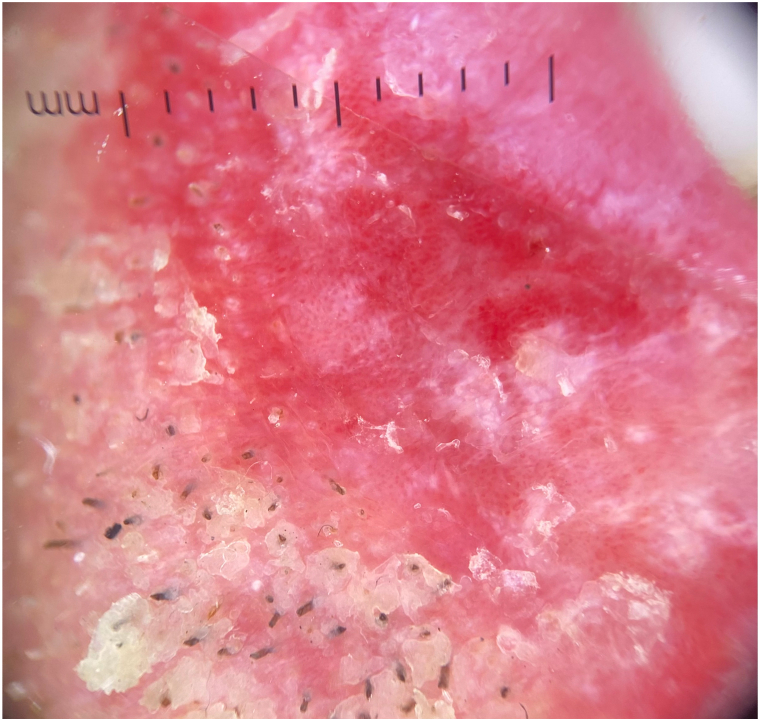
Dermoscopy of tinea labialis, demonstrating scaling and a lattice-like white network within central erosion.

**Fig 3:**
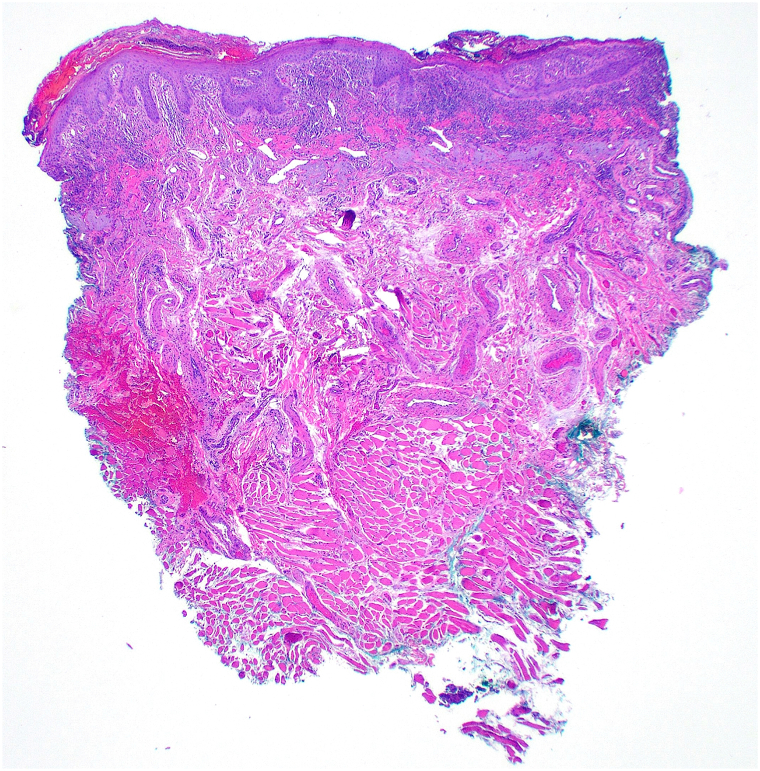
Histopathology of tinea labialis. Punch biopsy of tinea labialis showing focal epidermal erosion with inflammatory serum crust and mixed inflammation in the superficial dermis (hematoxylin and eosin, 20×).

**Fig 4:**
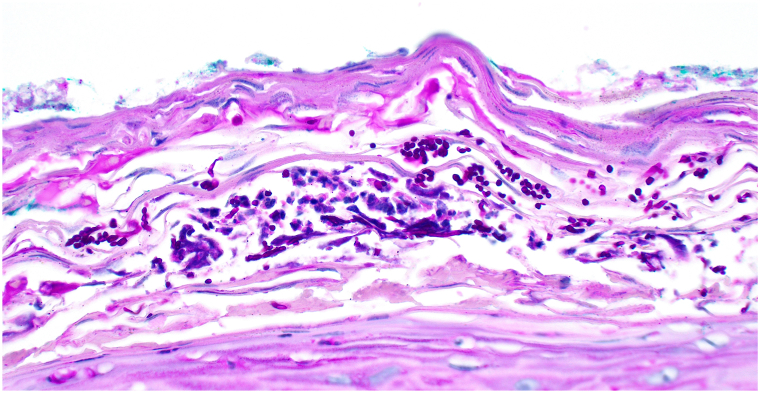
Fungal stain of tinea labialis. Histopathology of tinea labialis showing round yeast forms and refractile hyphae within the stratum corneum, admixed with neutrophils. Fungal elements stain *dark purple* and are mostly oriented horizontally (periodic acid Schiff stain, 400×).

A 62-year-old man with a history of SS,^4^ managed with anakinra for 2 years, presented to the dermatology clinic for the evaluation of swelling, pain, and scaling of his lower lip for months. He denied any trauma to the face but noted a recent sunburn that affected the lip. On examination, the lower vermillion lip had shallow erosions with adherent scaling at the periphery with some scaling to the cutaneous lips (Fig 1) and a white lattice-like structure within the erosions on dermoscopy (Fig 2). He had no known history of atopic dermatitis or seborrheic dermatitis. There was no appreciable intraoral involvement otherwise. A punch biopsy of the vermillion lip demonstrated epidermal erosion with hemorrhagic and inflammatory serum crust and a superficial perivascular mixed inflammatory infiltrate (Fig 3), with fungal hyphae and spores within the stratum corneum on periodic acid-Schiff stain (Fig 4). A diagnosis of tinea labialis was made. A fungal culture was performed, and the patient was started on weekly 200 mg fluconazole for 6 weeks, which resolved his symptoms. Weeks later, his symptoms recurred. The fungal culture eventually grew *Curvularia sp* and *Candida albicans*. The patient then started a prolonged 6-month course of weekly 200 mg fluconazole, which again resolved his symptoms, and he has since been under surveillance for this diagnosis.

## Discussion

The diagnosis of tinea labialis is exceedingly rare, with few cases reported in the literature.^5^ Our patient, who was receiving anakinra, presented with yeast and fungal hyphal elements on histopathology. Cultures grew both *Curvularia* (a mold) and *Candida* (a yeast), leading to a diagnosis of tinea labialis with concomitant candidiasis. *Curvularia* is a dematiaceous mold that rarely causes infection in immunocompetent individuals. We hypothesize that anakinra use contributed to the development of the *Curvularia* infection. The patient’s symptoms resolved with a 6-month course of 200 mg weekly fluconazole.

Anakinra functions by inhibiting IL-1 signaling, a pathway crucial for mucosal immune responses. IL-1 plays a pivotal role in activating phagocytes, inducing oxidative bursts, and upregulating defensins, all essential mechanisms for combating fungal pathogens on mucosal surfaces. Considering IL-1 plays a critical role in mucosal immunity,^6^ blocking it with anakinra may impair immune defenses and increase susceptibility to fungal infection. Additionally, IL-1 is integral to the differentiation of CD4+ T cells into Th17 cells, which produce cytokines like IL-17 and IL-22.^7^ These cytokines recruit neutrophils and help maintain epithelial integrity, forming a critical defense, particularly in the oral cavity, respiratory tract, and gastrointestinal system, against opportunistic pathogens such as *Candida albicans*. Consequently, by inhibiting IL-1, anakinra may diminish these vital mucosal defenses, increasing susceptibility to both fungal and yeast infections, as was observed in our patient. Additionally, IL-1 signaling appears to have a more substantial impact on mucosal immunity as compared to cutaneous immunity, particularly against fungal infections.^8^ At this time, increased risk of fungal infections is not associated with anakinra use. It is possible that our patient’s reported trauma of a sunburn injury allowed for fungal inoculation, and thus anakinra alone was not the sole contributor to his development of tinea labialis. Moreover, other treatment options for SS include canakinumab (an IL-1β antagonist) and rilonacept (IL-1α and IL-1β antagonist), neither of which has a strong association with fungal or yeast infections. Should our patient continue to have cutaneous fungal presentations, we would likely consider using canakinumab or rilonacept as an alternative treatment for SS.

This case highlights the rare diagnosis of tinea labialis and simultaneous infection of both *Candida albicans* and *Curvularia* in a patient on anakinra therapy. We hypothesize that ongoing use of an IL-1 receptor antagonist, in the absence of any other underlying conditions or medications, contributed to an impaired mucosal antifungal immune response, allowing for tinea labialis to occur. This case underscores the importance of recognizing fungal infections in patients receiving IL-1 inhibitors and of considering routine assessment of oral mucosa in patients on IL-1 inhibitors.

## Conflicts of interest

None disclosed.
